# The effect of obesity phenotype changes on cardiovascular outcomes in adults older than 40 years in the prospective cohort of the Tehran lipids and glucose study (TLGS): joint model of longitudinal and time-to-event data

**DOI:** 10.1186/s12889-024-18577-9

**Published:** 2024-04-23

**Authors:** Zahra Sedaghat, Soheila Khodakarim, Siamak Sabour, Majid Valizadeh, Maryam Barzin, Seyed Aria Nejadghaderi, Fereidoun Azizi

**Affiliations:** 1 Department of Epidemiology, School of Public Health and Safety, Shahid Beheshti University of Medical Sciences, Tehran, Iran; 2 Department of Biostatistics, School of Medicine, Shiraz University of Medical Sciences, Shiraz, Iran; 3 Safety Promotion and Injury Prevention Research Centre, Department of Epidemiology, School of Public Health and Safety, Shahid Beheshti University of Medical Sciences, Tehran, Iran; 4 Obesity Research Center, Research Institute for Endocrine Sciences, Shahid Beheshti University of Medical Sciences, Tehran, Iran; 5 HIV/STI Surveillance Research Center, WHO Collaborating Center for HIV Surveillance, Institute for Futures Studies in Health, Kerman University of Medical Sciences, Kerman, Iran; 6 Systematic Review and Meta-Analysis Expert Group (SRMEG), Universal Scientific Education and Research Network (USERN), Tehran, Iran; 7 Endocrine Research Center, Research Institute for Endocrine Sciences, Shahid Beheshti University of Medical Sciences, Tehran, Iran

**Keywords:** Obesity phenotypes, Cardiovascular diseases, Tehran lipid and glucose study, Myocardial infarction, Joint model, Binary outcome, Stroke

## Abstract

**Background:**

Obesity is a worldwide health concern with serious clinical effects, including myocardial infarction (MI), stroke, cardiovascular diseases (CVDs), and all-cause mortality. The present study aimed to assess the association of obesity phenotypes and different CVDs and mortality in males and females by simultaneously considering the longitudinal and survival time data.

**Methods:**

In the Tehran Lipid and Glucose Study **(**TLGS), participants older than three years were selected by a multi-stage random cluster sampling method and followed for about 19 years. In the current study, individuals aged over 40 years without a medical history of CVD, stroke, MI, and coronary heart disease were included. Exclusions comprised those undergoing treatment for CVD and those with more than 30% missing information or incomplete data. Joint modeling of longitudinal binary outcome and survival time data was applied to assess the dependency and the association between the changes in obesity phenotypes and time to occurrence of CVD, MI, stroke, and CVD mortality. To account for any potential sex-related confounding effect on the association between the obesity phenotypes and CVD outcomes, sex-specific analysis was carried out. The analysis was performed using packages (JMbayes2) of R software (version 4.2.1).

**Results:**

Overall, 6350 adults above 40 years were included. In the joint modeling of CVD outcome among males, literates and participants with a family history of diabetes were at lower risk of CVD compared to illiterates and those with no family history of diabetes in the Bayesian Cox model. Current smokers were at higher risk of CVD compared to non-smokers. In a logistic mixed effects model, odds of obesity phenotype was higher among participants with low physical activity, family history of diabetes and older age compared to males with high physical activity, no family history of diabetes and younger age. In females, based on the results of the Bayesian Cox model, participants with family history of diabetes, family history of CVD, abnormal obesity phenotype and past smokers had a higher risk of CVD compared to those with no history of diabetes, CVD and nonsmokers. In the obesity varying model, odds of obesity phenotype was higher among females with history of diabetes and older age compared to those with no history of diabetes and who were younger. There was no significant variable associated with MI among males in the Bayesian Cox model. Odds of obesity phenotype was higher in males with low physical activity compared to those with high physical activity in the obesity varying model, whereas current smokers were at lower odds of obesity phenotype than nonsmokers. In females, risk of MI was higher among those with family history of diabetes compared to those with no history of diabetes in the Bayesian Cox model. In the logistic mixed effects model, a direct and significant association was found between age and obesity phenotype. In males, participants with history of diabetes, abnormal obesity phenotype and older age were at higher risk of stroke in the Bayesian Cox model compared to males with no history of diabetes, normal obesity phenotype and younger persons. In the obesity varying model, odds of obesity phenotype was higher in males with low physical activity, family history of diabetes and older age compared to those with high physical activity, no family history of diabetes and who were younger. Smokers had a lower odds of obesity phenotype than nonsmokers. In females, past smokers and those with family history of diabetes were at higher risk of stroke compared to nonsmokers and females with no history of diabetes in the Bayesian Cox model. In the obesity varying model, females with family history of diabetes and older ages had a higher odds of obesity phenotype compared to those with no family history of diabetes and who were younger. Among males, risk of CVD mortality was lower in past smokers compared to nonsmokers in the survival model. A direct and significant association was found between age and CVD mortality. Odds of obesity phenotype was higher in males with a history of diabetes than in those with no family history of diabetes in the logistic mixed effects model.

**Conclusions:**

It seems that modifications to metabolic disorders may have an impact on the heightened incidence of CVDs. Based on this, males with obesity and any type of metabolic disorder had a higher risk of CVD, stroke and CVD mortality (excluding MI) compared to those with a normal body mass index (BMI) and no metabolic disorders. Females with obesity and any type of metabolic disorder were at higher risk of CVD(, MI and stroke compared to those with a normal BMI and no metabolic disorders suggesting that obesity and metabolic disorders are related. Due to its synergistic effect on high blood pressure, metabolic disorders raise the risk of CVD.

**Supplementary Information:**

The online version contains supplementary material available at 10.1186/s12889-024-18577-9.

## Introduction

Obesity is a worldwide health concern with serious clinical effects, including myocardial infarction (MI), stroke, cardiovascular diseases (CVDs), and all-cause mortality [1]. More than 650 million adults are reported to be obese worldwide. More worryingly, the prevalence of obesity has tripled between 1957 and 2016 [2]. In the Middle East and North Africa region, there was a non-significant increase in the burden and deaths attributable to excess body weight over the last three decades [3]. Results of a systematic review showed that the prevalence of overweight and obesity was above 35% in the total population [4]. It is believed that a collection of symptoms known as metabolic syndrome is contributing to CVD [5]. Metabolic syndrome is a disorder defined by the co-occurrence of at least three out of five medical conditions, namely elevated blood pressure, high blood sugar, elevated triglycerides, low density of lipoprotein, and obesity [6].

Not all patients with obesity suffer from metabolic disorders, this syndrome can also occur in normal-weight individuals, known as metabolically unhealthy normal weight (MUNW) [2]. Although much evidence demonstrates that obesity and metabolic abnormalities are major risk factors for CVD, information about the nature of the association is limited [5]. Previous prospective cohort studies have indicated that metabolically healthy obese patients are at higher risk of CVD and mortality than metabolically healthy normal-weight individuals [7, 8]. However, there are some observational studies that do not support the association between obesity and CVD [9, 10].

Since obesity and its complications are one of the major public health concerns, numerous studies have been conducted to investigate the association between obesity phenotypes and the incidence of CVD or the attributable mortalities [11, 12]. Most of previous studies have used the survival analysis or linear mixed effect models separately [1, 9, 10], while they have not considered the association between longitudinal process, changes in obesity phenotypes, and time-to-occur CVD. Consequently, we aimed to assess the association of obesity phenotypes on CVDs by simultaneously considering the longitudinal and survival time data in order to increase the efficiency of the estimate of the longitudinal markers and prevent probable biases.

## Methods

### Study design and setting

The Tehran Lipid and Glucose Study (TLGS) is a prospective population-based study which was conducted to determine risk factors for non-communicable diseases in a representative urban Tehran population. The TLGS was performed in six phases: phase one from 1999 to 2000, phase two from 2001 to 2004, phase three from 2005 to 2007, phase four from 2008 to 2010, phase five from 2011 to 2014, and phase six from 2015 to 2018. In the TLGS, 15,005 participants aged over three years were selected by multi-stages random cluster sampling method [13]. This study included 6,350 of participants over 40 years who followed for approximately 19 years. The current study was approved by the ethics committee of Shahid Beheshti University of Medical Sciences, Tehran, Iran (ethics code: IR.SBMU.PHNS.REC.1400.159). The study was conducted in accordance with the principles of the Declaration of Helsinki and the national guidelines and regulations.

### Study population

We included participants aged over 40 years without a medical history of CVDs including stroke, MI, and coronary heart disease at baseline. Individuals who did not give informed consent to participate, those who received treatment for CVD, and those with more than 30% missing information or incomplete data were excluded.

### Data collection

An interview was conducted to collect data on past medical history, family history, smoking status, and physical activity in each phase. The questionnaire consisted of questions regarding demographic information (age, sex, and education level), physical activity (sport and job-related), and medical history (i.e., family, habitual, and drug history). Anthropometric measures (i.e., weight, height, waist circumference (WC), and hip circumferences (HC)), systolic blood pressure (SBP), and diastolic blood pressure (DBP) were measured by a trained nurse. Blood samples were taken based on the standard protocols for measuring blood biomarkers, including fasting blood sugar (FBS), low-density lipoprotein (LDL), high-density lipoprotein (HDL), and triglyceride (TG). All these variables were measured and recorded in each phase.

### Definitions

Metabolically unhealthy condition was defined using the criteria proposed by the Joint Interim Statement (JIS) [9] as follows: (1) FBS ≥ 100 mg/dl (5.5 mmol/l) or 2-h blood glucose ≥ 140 mg/dl (7.8 mmol/l) or drug treatment; (2) fasting TGs ≥ 150 mg/dl (1.7 mmol/l) or drug treatment; (3) fasting HDL-C < 50 mg/dl (1.3 mmol/l) in women and < 40 mg/dl (1.0 mmol/l) in men or drug treatment; (4) raised blood pressure defined as SBP ≥ 130 mmHg, DBP ≥ 85 mmHg or antihypertensive drug treatment; and (5) WC > 89 cm for men and > 91 cm for women based on national cut-offs [9]. The metabolically healthy condition was considered to have none or only one component of the JIS, and participants with two or more criteria were considered asmetabolically unhealthy. BMI was calculated as the weight (kg) divided by height squared (m^2^) and was categorized into two groups: those with a BMI below 25 kg/m² were classified as normal BMI and those with a BMI of 25 kg/m² or higher were considered obese.

In this regard, four categories were developed:

- Metabolically healthy normal weight (MHNW): Those participants with normal BMI and none or only one sign of metabolic syndrome.
- Metabolically unhealthy normal weight (MUNW): Those with normal BMI and with at least two signs of metabolic syndrome.
- Metabolically healthy obesity (MHO): Participants with none or only one sign of metabolic syndrome and obesity.
- Metabolically unhealthy obesity (MUO): Those with obesity and with at least two signs of metabolic syndrome.

Also, CVD defined as time to occurrence of any CVD. Stroke was defined as time to occurrence of new neurological deficit lasting more than 24 h. MI as the time to occurrence of a diagnostic electrocardiogram (ECG) and biomarker results. CVD mortality was defined as the time to occurrence of any death attributed to CVD.

### Statistical analysis

Multiple imputation was applied to participants with missing, unrecorded, or incomplete information at each phase. The MICE package in R was applied for imputation. Imputation was applied five times for the purpose of detecting a more consistent dataset for non-attributed data. Sensitivity analysis was done based on hazard ratio (HR) and p-value. Next, the univariate analysis was performed to determine the association of each covariate (demographic variables, physical activity, smoking, and medical history of participants and their families) with the interest outcomes. Regarding that, datasets with high concurrence owing to non-imputed data were selected as underlying data.

The primary study variables to describe participants’ characteristics in the baseline included both quantitative measures including age, SBP, DBP, FBS, TG, LDL, HDL, cholesterol, BMI, and qualitative measures including sex, level of education, physical activity, obesity phenotypes, smoking status, family history of diabetes and family history of CVD. In our study, four categories of obesity phenotypes were combined into two groups which were the normal group (those with normal BMI and healthy metabolic status) and the abnormal group (those with abnormality either in their BMI or their metabolic status) for easy and comprehensible interpretation. Time to occurrence of CVD, stroke, MI, and mortalities attributable to CVD were considered as response variables and outcomes of interest.

To report the main characteristics of the study population, (mean ± standard deviation [SD]) and (n, %) were used for quantitative and qualitative variables, respectively. The joint model of longitudinal binary measurements and survival time data was applied to combine information from both types of data to provide a comprehensive analysis. Longitudinal binary measurements, obesity phenotypes measured in the six phases of the TLGS study, and survival times, times until occur CVD, MI, stroke, and CVD mortality, were collected on the same individuals over time.

The joint model consists of two sub-models which were a longitudinal sub-model and a survival sub-model. The survival incorporates the longitudinal measurements as time-varying covariates, allowing the association between longitudinal and survival outcomes to be examined.

A logistic mixed effects model was used to investigate the association between age, sex, smoking status, physical activity, family history of CVD, family history of diabetes and the obesity phenotype measured across six time points. This model accounts for the individual-specific variation in the obesity phenotypes over time. The results of the Bayesian logit mixed effect model were reported as odds ratio (OR) and 95% credible interval (95% CI) (Appendix 1).

A Bayesian Cox model was used to evaluate the association between age, sex, education, smoking status, physical activity, family history of CVD, family history of diabetes, obesity phenotypes and the time to occurrence of CVD, MI, stroke, and CVD mortality. The results of the Bayesian Cox model and the survival model were reported as hazard ratio (HR) and 95% CIs. The joint model was applied only for outcomes where the α parameter indicated that the association between longitudinal and time to the event model was statistically significant. If the α parameter was not statistically significant, a Bayesian Cox model and a logistic mixed model were applied separately. To account for any potential sex-related confounding effect on the association between the obesity phenotypes and CVD outcomes, sex-specific analysis was carried out. The analysis was performed using packages (JMbayes2) of R software (version 4.2.1).

## Results

We included 6,350 participants over age 40 years who were predominantly female (54.5%) with a mean age of 54.3 (SD: 10.1) years. Also, most participants were literate (68.4%) and nonsmokers (50.6%). The mean BMI was 27.9 kg/m2 (SD: 4.6 kg/m2) and 45.6% had metabolically unhealthy obesity. The baseline mean FBS and cholesterol levels were 196.8 (SD: 130.7) mg/dl and 225.4 (SD: 47.3) mg/dl, respectively, with normal SBP (127.6; SD: 21.5 mmHg) and DBP (80.7; SD: 11.4 mmHg) (Table 1).

Obesity trends in participants included: 48.7% changed from normal to obesity status and only 4.90% changed from obesity to normal status in phase 2. In phase 3, 34.7% of participants switched from normal to obesity status and only 4.7% switched from obesity to normal status. Moreover, 44.1% switched from normal to obesity status and only 4.1% of participants switched from obesity to normal status in phase 4. In phase 5, 39.8% of participants switched from normal to obesity status and only 4.2%s switched from obesity to normal status. In addition, 42.9% of participants switched from normal to obesity status and 3.9% switched from obesity to normal status in phase 6 (Table 2).

**Table 1:** Baseline characteristics of participants

		Total (*n*=6350)
		**N(%)**
Sex	Male	2892(45.5)
	Female	3458(54.5)
Education	Illiterate	966(15.2)
	Literate	4345(68.4)
	Non response	1039(16.4)
History of diabetes	No	3539(55.7)
	Yes	1519(23.9)
	Non response	1292(20.3)
Family history of CVD	No	4329(68.2)
	Yes	906(14.3)
	Non response	1115(17.6)
Smoking status	Current smoker	434(6.8)
	Past smoker	451(7.1)
	Never	3211(50.6)
	Non response	2254(35.5)
Physical activity	Highest	2680(42.2)
	Lowest	1647(25.9)
	Non response	2023(31.9)
Obesity phenotypes	MHNW	591(9.3)
	MUNW	740(11.6)
	MHO	862(13.6)
	MUO	2897(45.6)
	Non-response	1260(19.8)
		**Mean±SD**
Age (year)		54.3±10.1
Systolic blood pressure (mmHg)		127.7±21.5
Diastolic blood pressure (mmHg)		80.8±11.4
FBS (mg/dl)		106.9±42.1
TG (mg/dl)		196.8±130.7
LDL (mg/dl)		144.9±38.7
HDL (mg/dl)		42.1±11.0
Cholesterol (mg/dl)		225.4±47.2
BMI (kg/m2)		27.9±4.5

**Table 2:** Changes of obesity status of participants in different phases of the Tehran lipid and glucose study

Phases	Trend of obesity	Total
		Percent
Phase 1	Normal	12.6
	Abnormal	87.3
Phase 2	Normal to Abnormal	48.7
	Abnormal to Normal	4.9
	Normal to Normal	51.3
	Abnormal to Abnormal	95.1
Phase 3	Normal to Abnormal	34.7
	Abnormal to Normal	4.7
	Normal to Normal	65.3
	Abnormal to Abnormal	95.3
Phase 4	Normal to Abnormal	44.1
	Abnormal to Normal	4.1
	Normal to Normal	55.9
	Abnormal to Abnormal	95.9
Phase 5	Normal to Abnormal	39.8
	Abnormal to Normal	4.2
	Normal to Normal	60.2
	Abnormal to Abnormal	95.8
Phase 6	Normal to Abnormal	42.9
	Abnormal to Normal	3.9
	Normal to Normal	57.1
	Abnormal to Abnormal	96.1

The highest incidence of CVD among participants occurred in phase 2 (6.93%). The highest incidence of MI occurred in phase 2 (2.15%). The highest incidence of stroke occurred in phase 5 (1.42%). The highest incidence of CVD mortality occurred in phase 2 (6.53%) (Appendix 2).

According to the results from sex-specific analysis, joint model for CVD outcome among males showed literates (HR _literate/illiterate_=0.69, 95% CI: 0.56 to 0.87, *P* = 0.002) and participants with family history of diabetes (HR _yes/no_ = 0.75, 95% CI: 0.58 to 0.95, *P* = 0.01) were at lower risk of CVD compared to illiterates and those with no family history of diabetes in the Bayesian Cox model. While, current smokers (HR _current smoker/nonsmoker_ = 1.37, 95% CI: 1.07 to 1.80, *P* = 0.01) were at higher risk of CVD compared to non-smokers. In the logistic mixed effects model, odds of obesity phenotype was higher among participants with low physical activity (OR _lowest/highest_= 1.52, 95% CI: 1.06 to 2.05, *P* = 0.02), family history of diabetes (OR _yes/no_ = 3.42, 95% CI: 2.36 to 5.25, *P* < 0.001) and older age (OR = 1.05, 95% CI: 1.04 to 1.05, *P* < 0.001) compared to males with high physical activity, no family history of diabetes and younger age. While, current smokers (OR _current smoker/ nonsmoker_ = 0.26, 95% CI: 0.18 to 0.37, *P* < 0.001) were at lower odds of obesity phenotype compared to nonsmokers. In females, those with a family history of diabetes (HR _yes/no_ = 1.51, 95% CI: 1.29 to 1.77, *P* < 0.001), a family history of CVD (HR _yes/no_ = 1.42, 95% CI: 1.18 to 1.71, *P* < 0.001), abnormal obesity phenotype (HR _abnormal/normal_ = 2.29, 95% CI: 1.48 to 3.54, *P* < 0.001), older age (HR = 1.05, 95% CI: 1.05 to 1.07, *P* < 0.001) and past smokers (OR _past smoker/nonsmoker_ =1.80, 95% CI: 1.22 to 2.66, *P* = 0.002) had a higher risk of CVD compared to those with no history of diabetes or CVD, younger age and nonsmokers in the Bayesian Cox model. Risk of CVD was lower among literate females compared to illiterates (HR _literate/illiterate_ =0.78, 95% CI: 0.64 to 0.94, *P* = 0.009). In the obesity varying model, odds of obesity phenotype was higher among females with history of diabetes (OR _yes/no_ = 1.67, 95% CI: 1.06 to 2.63, *P* = 0.02) and older age (OR = 1.13, 95% CI: 1.12 to 1.15, *P* < 0.001) compared to those with no history of diabetes and younger age (Table 3).

**Table 3:** Effects of time varying obesity phenotypes and baseline covariates on the incidence of CVD among males using the joint modelling approach

Male (2892)							
CVD from Bayesian Cox model					Obesity varying from Bayesian logit mixed effects model		
Variables		Mean (SD)	HR (95% CI)	P-value	Mean (SD)	OR (95% CI)	P-value
Education (Reference: illiterate)	Literate	-0.36 (0.11)	0.69 (0.56 to 0.87)	0.002	-	-	-
Smoking status (Reference: Never smoker)	Past smoker	0.38 (0.10)	1.46 (1.18 to 1.80)	<0.001	-0.49 (0.21)	0.61 (0.39 to 0.95)	0.02
	Current smoker	0.32 (0.13)	1.37 (1.07 to 1.80)	0.01	-1.33 (0.18)	0.26 (0.18 to 0.37)	<0.001
Physical activity (Reference: Highest)	Lowest	-0.15 (0.08)	0.86 (0.72 to 1.01)	0.07	0.42 (0.17)	1.52 (1.06 to 2.05)	0.02
Family history of diabetes (Reference: No)	Yes	-0.28 (0.12)	0.75 (0.58 to 0.95)	0.01	1.23 (0.20)	3.42 (2.36 to 5.25)	<0.001
Family history of CVD (Reference: No)	Yes	0.10 (0.11)	1.10 (0.89 to 1.37)	0.35	0.04 (0.23)	1.04 (0.67 to 1.63)	0.87
Obesity phenotypes (Reference: Normal)	Abnormal	0.05 (0.17)	1.05 (0.76 to 1.49)	0.76	-	-	-
Age (year)		-0.13 (0.26)	0.87 (0.49 to 1.29)	0.63	0.05 (0.004)	1.05 (1.04 to 1.06)	<0.001
**The associations of study variables with incidence of CVD among females using survival and longitudinal models**							
**Female (3458)**							
**CVD from Bayesian Cox model**					**Obesity varying from Bayesian logit mixed effects model**		
**Variables**		**HR (95% CI)**		**P-value**	**OR (95% CI)**		**P-value**
Education (Reference: illiterate)	Literate	0.78 (0.64 to 0.94)		0.009	-		-
Smoking status (Reference: Never smoker)	Past smoker	1.80 (1.22 to 2.66)		0.002	1.27 (0.28 to 5.58)		0.75
	Current smoker	0.97 (0.54 to 1.72)		0.92	0.43 (0.17 to 1.08)		0.07
Physical activity (Reference: Highest)	Lowest	1.08 (0.92 to 1.27)		0.29	1.05 (0.70 to 1.55)		0.80
Family history of diabetes (Reference: No)	Yes	1.51 (1.29 to 1.77)		<0.001	1.67 (1.06 to 2.63)		0.02
Family history of CVD (Reference: No)	Yes	1.42 (1.18 to 1.71)		<0.001	1.84 (0.99 to 3.45)		0.05
Obesity phenotypes (Reference: Normal)	Abnormal	2.29 (1.48 to 3.54)		<0.001	-		-
Age (year)		1.05 (1.05 to 1.06)		<0.001	1.13 (1.12 to 1.15)		<0.001

α: association between survival model and longitudinal model, p-value<0.001

*Abbreviations* CVD: cardiovascular disease; HR: hazard ratio; OR: odds ratio; CI: credible interval

There was no significant variable associated with MI among males in the Bayesian Cox model. Odds of obesity phenotype was higher in males with low physical activity compared to those with high physical activity in the obesity varying model (OR _lowest/highest_ =1.63, 95% CI: 1.18 to 2.27, *P* = 0.002). Current smokers were at lower odds of obesity phenotype than nonsmokers (OR _current smoker/nonsmoker_ =0.27, 95% CI: 0.18 to 0.40, *P* < 0.001). In females, risk of MI was higher among those with a family history of diabetes (HR _yes/no_= 1.81, 95% CI: 1.29 to 2.54, *P* < 0.001) and older age (HR = 1.08, 95% CI: 1.06 to 1.10, *P* < 0.001) compared to females with no history of diabetes and younger age in the Bayesian Cox model. In logistic mixed effects model, a direct and significant association was found between age and obesity phenotype (OR = 1.15, 95% CI: 1.13 to 1.71, *P* < 0.001) (Table 4).

**Table 4:** Effects of time varying obesity phenotypes and baseline covariates on the incidence of MI among males using the joint modelling approach

Male (2892)							
MI from Bayesian Cox model					Obesity varying from Bayesian logit mixed effects model		
Variables		Mean (SD)	HR (95% CI)	P-value	Mean (SD)	OR (95% CI)	P-value
Education (Reference: illiterate)	Literate	-0.31 (0.19)	0.73 (0.50 to 1.08)	0.12	-	-	-
Smoking status (Reference: Never smoker)	Past smoker	-0.10 (0.17)	0.90 (0.65 to 1.24)	0.55	-0.43 (0.22)	0.65 (0.42 to 0.99)	0.05
	Current smoker	0.22 (0.17)	1.24 (0.90 to 1.75)	0.19	-1.30 (0.19)	0.27 (0.18 to 0.40)	<0.001
Physical activity (Reference: Highest)	Lowest	0.02 (0.12)	1.02 (0.79 to 1.29)	0.84	0.49 (0.16)	1.63 (1.18 to 2.27)	0.002
Family history of diabetes (Reference: No)	Yes	-0.18 (0.14)	0.83 (0.62 to 1.09)	0.21	-	-	-
Family history of CVD (Reference: No)	Yes	-0.13 (0.18)	0.87 (0.61 to 1.23)	0.48	-	-	-
Obesity phenotypes (Reference: Normal)	Abnormal	-0.45 (0.39)	0.63 (0.28 to 1.35)	0.24	-	-	-
Age (year)		0.08 (0.21)	1.08 (0.79 to 1.69)	0.87	0.01 (0.01)	1.01 (0.99 to 1.03)	0.13
**The associations of study variables with incidence of MI among females using survival and longitudinal models**							
**Female (3458)**							
**MI from Bayesian Cox model**					**Obesity varying from Bayesian logit mixed effects model**		
**Variables**		**HR (95% CI)**		**P-value**	**OR (95% CI)**		**P-value**
Education (Reference: illiterate)	Literate	0.71 (0.48 to 1.04)		0.08	-		-
Smoking status (Reference: Never smoker)	Past smoker	0.85 (0.27 to 2.68)		0.78	1.15 (0.29 to 4.60)		0.83
	Current smoker	1.23 (0.39 to 3.89)		0.71	0.44 (0.16 to 1.15)		0.09
Physical activity (Reference: Highest)	Lowest	1.001 (0.70 to 1.41)		0.99	1.05 (0.70 to 1.56)		0.80
Family history of diabetes (Reference: No)	Yes	1.81 (1.29 to 2.54)		<0.001	-		-
Family history of CVD (Reference: No)	Yes	1.35 (0.91 to 2.01)		0.13	-		-
Obesity phenotypes (Reference: Normal)	Abnormal	2.56 (0.94 to 6.91)		0.06	-		-
Age (year)		1.08 (1.06 to 1.10)		<0.001	1.15 (1.13 to 1.71)		<0.001

α: association between survival model and longitudinal model, p-value<0.001

*Abbreviations* CVD: cardiovascular disease; SD: standard deviation; HR: hazard ratio; OR: odds ratio; CI: credible interval

Regarding the results of the Bayesian Cox model in males, participants with a history of diabetes (HR _yes/no_= 1.62, 95% CI: 1.17 to 2.25, *P* = 0.003), abnormal obesity phenotype (HR _abnormal/normal_ = 1.56, 95% CI: 1.01 to 2.41, *P* = 0.04) and older age (HR = 1.09, 95% CI: 1.07 to 1.11, *P* < 0.001) were at higher risk of stroke compared to males with no history of diabetes, normal obesity phenotype and younger age. In the obesity varying model, odds of obesity phenotype was higher in males with low physical activity (OR _lowest/highest_ =1.62 95% CI: 1.18 to 2.21, *P* = 0.002), family history of diabetes (OR _yes/no_ = 3.09, 95% CI: 2.12 to 4.52, *P* < 0.001) and older age (OR = 1.03, 95% CI: 1.01 to 1.05, *P* < 0.001) compared to those with high physical activity, no family history of diabetes and younger age. Smokers had a lower odds of obesity phenotype than nonsmokers (OR _current smoker/nonsmoker_ =0.30, 95% CI: 0.20 to 0.44, *P* < 0.001). In females, past smokers (HR _past smoker/nonsmoker_ =2.59, 95% CI: 1.31 to 5.13, *P* = 0.005), those with family history of diabetes (HR _yes/no_ = 1.46, 95% CI: 1.04 to 2.05, *P* = 0.02), and older participants (HR = 1.09, 95% CI: 1.06 to 1.11, *P* < 0.001) were at higher risk of stroke compared to nonsmokers, females with no history of diabetes and younger age in the Bayesian Cox model. On the other hand, literates (HR _literate/illiterate_=0.50, 95% CI: 0.34 to 0.74, *P* < 0.001) had a lower risk of stroke than illiterates. In the obesity varying model, females with a family history of diabetes (OR _yes/no_ = 1.61, 95% CI: 1.02 to 2.55, *P* = 0.03) and older ages (OR = 1.17, 95% CI: 1.15 to 1.18, *P* < 0.001) had a higher odds of obesity phenotype compared to those with no family history of diabetes and younger age (Table 5).

**Table 5:** The associations of study variables with incidence of stroke using survival and longitudinal models stratified by sex

Stroke from Bayesian Cox model					
		Male(2892)		Female (3458)	
Variables		HR (95% CI)	P-value	HR (95% CI)	P-value
Education (Reference: illiterate)	Literate	1.20 (0.77 to 1.87)	0.40	0.50 (0.34 to 0.74)	<0.001
Smoking status (Reference: Never smoker)	Past smoker	1.18 (0.82 to 1.71)	0.35	2.59 (1.31 to 5.13)	0.005
	Current smoker	1.35 (0.87 to 2.08)	0.17	1.39 (0 to inf)	0.99
Physical activity (Reference: Highest)	Lowest	0.88 (0.65 to 1.19)	0.43	1.22 (0.87 to 1.71)	0.24
Family history of diabetes (Reference: No)	Yes	1.62 (1.17 to 2.25)	0.003	1.46 (1.04 to 2.05)	0.02
Family history of CVD (Reference: No)	Yes	1.09 (0.70 to 1.68)	0.69	1.39 (0.94 to 2.06)	0.09
Obesity phenotypes (Reference: Normal)	Abnormal	1.56 (1.01 to 2.41)	0.04	2.68 (0.98 to 7.27)	0.05
Age (year)		1.09 (1.07 to 1.11)	<0.001	1.09 (1.06 to 1.11)	<0.001
**Obesity varying from Bayesian logit mixed effects model**					
		**Male (2892)**		**Female (3458)**	
**Variables**		**OR (95% CI)**	**P-value**	**OR (95% CI)**	**P-value**
Education (Reference: illiterate)	Literate	-	-	-	-
Smoking status (Reference: Never smoker)	Past smoker	0.63 (0.41 to 0.96)	0.03	1.15 (0.26 to 5.07)	0.85
	Current smoker	0.30 (0.20 to 0.44)	<0.001	0.43 (0.16 to 1.16)	0.09
Physical activity (Reference: Highest)	Lowest	1.62 (1.18 to 2.21)	0.002	1.02 (0.68 to 1.53)	0.88
Family history of diabetes (Reference: No)	Yes	3.09 (2.12 to 4.52)	<0.001	1.61 (1.02 to 2.55)	0.03
Family history of CVD (Reference: No)	Yes	1.006 (0.63 to 1.59)	0.97	1.73 (0.93 to 3.21)	0.08
Obesity phenotypes (Reference: Normal)	Normal	-	-	-	-
Age (year)		1.03 (1.01 to 1.05)	<0.001	1.17 (1.15 to 1.18)	<0.001

*Abbreviations* CVD: Cardiovascular disease; CI: credible interval; HR: hazard ratio; OR: odds ratio

Among males, risk of CVD mortality was lower in past smokers compared to nonsmokers in the survival model (HR _past smoker/nonsmoker_= 0.56, 95% CI: 0.38 to 0.83, *P* = 0.003). However, a direct and significant association was found between age and CVD mortality (HR = 1.08, 95% CI: 1.06 to 1.10, *P* < 0.001). Odds of obesity phenotype was higher in males with a history of diabetes than those with no family history of diabetes in the logistic mixed effects model (OR _yes/no_ = 2.96, 95% CI: 1.54 to 5.69, *P* = 0.001). Current smokers had a lower odd of obesity phenotype than nonsmokers (OR _current smoker/nonsmoker_ =0.40, 95% CI: 0.20 to 0.79, *P* = 0.008). Because CVD mortality in women was rare, we were unable to apply the logistic mixed effects model to that outcome in females (Table 6).

**Table 6:** The associations of study variables with incidence of CVD mortality using survival and longitudinal models stratified by sex

CVD mortality from Bayesian Cox model					
		Male (2892)		Female (3458)	
Variables		HR (95% CI)	P-value	HR (95% CI)	P-value
Education (Reference: illiterate)	Literate	0.91 (0.62 to 1.33)	0.64	-	-
Smoking status (Reference: Never smoker)	Past smoker	0.56 (0.38 to 0.83)	0.003	-	-
	Current smoker	1.37 (0.95 to 1.98)	0.08	-	-
Physical activity (Reference: Highest)	Lowest	1.06 (0.81 to 1.39)	0.63	-	-
Family history of diabetes (Reference: No)	Yes	1.18 (0.86 to 1.60)	0.29	-	-
Family history of CVD (Reference: No)	Yes	1.01 (0.68 to 1.50)	0.95	-	-
Obesity phenotypes (Reference: Normal)	Abnormal	1.16 (0.78 to 1.73)	0.44	-	-
Age (year)		1.08 (1.06 to 1.10)	<0.001	-	-
**Obesity varying from Bayesian logit mixed effects model**					
		**Male (2892)**		**Female (3458)**	
**Variables**		**OR (95% CI)**	**P-value**	**OR (95% CI)**	**P-value**
Education (Reference: illiterate)	Literate	-	-	-	-
Smoking status (Reference: Never smoker)	Past smoker	0.66 (0.35 to 1.24)	0.20	-	-
	Current smoker	0.40 (0.20 to 0.79)	0.008	-	-
Physical activity (Reference: Highest)	Lowest	1.17 (0.70 to 1.96)	0.53	-	-
Family history of diabetes (Reference: No)	Yes	2.96 (1.54 to 5.69)	0.001	-	-
Family history of CVD (Reference: No)	Yes	0.90 (0.43 to 1.89)	0.79	-	-
Obesity phenotypes (Reference: Normal)	Normal	-	-	-	-
Age (year)		0.98 (0.95 to 1.01)	0.42	-	-

*Abbreviations* CVD: Cardiovascular disease; CI: credible interval; HR: hazard ratio; OR: odds ratio

## Discussion

Men and women differed across the incidence and progression of CVD outcomes, which could be related to obesity, metabolic status, and sexual hormones. We therefore carried out a sex- specific analysis in order to control for such differences. Overall, our findings on the association between obesity phenotypes and CVD outcomes showed that smoking and abnormal obesity phenotype increase the risk of CVD in both males and females. Family history of diabetes and age were associated with increased risk of MI in females but not in males. Furthermore, abnormal obesity phenotype, age and family history of diabetes were directly associated with stroke in both males and females. However, in terms of CVD mortality, past smoking significantly decreased the risk of CVD mortality. In the obesity varying model, low physical activity, family history of diabetes and age were directly and significantly associated with obesity phenotype in males. Current smoking decreased the odds of obesity phenotype in males and females. In females, only family history of diabetes and age were directly associated with obesity phenotype.

According to the results of the joint modeling for CVD, individuals with obesity or metabolic disorders were at higher risk of CVD than those with normal BMI and without metabolic disorders. It seems that obese individuals are at higher risk of metabolic disorders such as hypertension and hyperlipidemia, which are thought to play an important role in the causation of CVD [14]. The obesity phenotype variation model in males indicates that changes in obesity phenotypes over a two-decade follow-up period is a major risk factor for CVD. Although this study did not find any significant association between the longitudinal and time to occur of CVD model in females, an abnormal obesity phenotype increased the risk of CVD approximately two-fold in women. Regarding the results of the Bayesian Cox model among females, our study suggests that the risk of CVD was more common among current and past smokers. In this regard, the article by Keto et al. showed that smokers compared with nonsmokers had higher levels of cholesterol, LDL, TG and lower levels of HDL [15]. Based on the results of the logistic mixed model, males with low physical activity, a family history of diabetes and older age were at higher odds of having the obesity phenotype. One of the interesting findings of the present study among both males and females was that smokers had a lower odds of abnormal obesity phenotype than non-smokers. This result could be related to higher metabolism and lower energy intake in smokers than non-smokers [16]. The present study also suggests that the risk of CVD was more common among male past smokers. Accordingly, the study by Amiri et al. on a sample of the TLGS showed an increased risk of CVD in daily and occasional male smokers [17]. This finding could be due to the various effects of smoking, including vasomotor dysfunction and atherogenesis.

In this study, different obesity phenotypes accounted for the greatest proportions of CVD, stroke, and MI incidence. This finding is consistent with studies by Zheng et al. and Popa et al., who reported that metabolic changes were more important risk factors than obesity in increasing the risk of CVD outcomes [18, 19]. In this regard, individuals with obesity were more likely to develop an unhealthy metabolic state and have an increased risk of developing CVD. However, our findings show that there is a protective role of obesity for the incidence of MI among males. This can be explained by several mechanisms. First, a concept called the “obesity paradox” suggests that excess body weight can lead to a better prognosis for CVD [20]. Second, an in vitro study showed that resistin, a hormone secreted by adipose tissue, can also play a protective role against MI [21]. Additional observational studies on humans are recommended to further evaluate the effects of obesity on CVD outcomes, especially MI among men.

It has been suggested that increased physical activity could play an important role in the reduction of both obesity and unhealthy metabolic status in both males and females [22]. Another important finding of this study was that females with a family history of diabetes were at higher risk of MI, CVD and stroke which was also reported by Akhuemonkhan et al. [23] and Christie et al. [24]. However, a family history of diabetes in men was associated with reduced risk of CVD. In this regard, a cohort study on Korean subjects who did not have diabetes showed that a family history of diabetes was associated with increased risk of ischemic heart disease, while it did not significantly increase the risk of cerebrovascular disease or atherosclerotic CVD [25].

In this study the α parameter was not significant for the CVD, and MI models in females, CVD mortality in males and also stroke in both males and females. Based on the Bayesian Cox model, obesity phenotypes increased the risk of stroke in both males (1.56 times) and especially females (2.68 times). This finding is consistent with the results of studies published by Lee et al. [26] and Laura et al. [27]. Obesity increases the risk of stroke among those with unhealthy metabolic status compared to those with a healthy metabolic group, possibly due to the important role of the metabolic syndrome [26, 27]. One possible explanation for this finding is that the biological components of the metabolic syndrome, including hyperglycemia, hyperlipidemia, and especially hypertension, also independently play important roles in the development of stroke [28]. According to the logistic mixed model, current smokers of either sex were less likely to have obesity or the metabolic syndrome. This result is consistent with the study by Khodamoradi and colleagues [29]. It seems that smokers due to change in eating habits mainly due to olfactory and appetite disorders, have less food preference [16].

One of the interesting findings of this study is that non-smokers had a higher risk of MI than past smokers which is consistent with other previous studies [30, 31]. It might be due to an improvement of numerous pathophysiological processes including plasma fibrinogen, reactive capillary flow, and transcutaneous partial oxygen tension [32]. Additionally, other CVD risk factors such as hematocrit and white blood cell count also exhibited greater reductions in abstainers. Furthermore, arterial stiffness which is an indicator of hypertension showed a significant reduction after smoking cessation [33]. Interestingly, CVD mortality was also lower among past smokers than non-smokers. It seems that because of rapid onset of symptoms in smokers and increased number of visits to physicians, CVD are diagnosed earlier in those people, which may reduce the risk of death. In addition, past smokers may change their habits due to the advice given to them by physicians and this behavioral adjustment would increase their survival compared to non-smokers.

A strength of our study is the use of both longitudinal and survival time data simultaneously to determine the effects of obesity as one of the major risk factors for different CVD outcomes. We evaluated different obesity phenotype groups compared to people with normal BMI. We examined groups with both healthy and unhealthy metabolic status. Using these data, we were able to determine the relationship between BMI status and different types of CVD using joint model for outcomes with significant α parameter. The results showed that changes in BMI, regardless of metabolic status, were significantly associated with the incidence of stroke and CVD. Then, in another model, metabolically unhealthy individuals were compared with metabolically healthy individuals, irrespective of obesity, in order to determine associations with different types of cardiovascular disease. According to the results of the joint model, changes in metabolic status, independent of obesity, were significantly associated with stroke, CVD, and MI.

It can be concluded that changes in BMI, independent of metabolic status, play an important role in increasing the risk of stroke compared to the other two types of obesity. Thus, people with obesity, regardless of their metabolic status, are at about 4 times higher risk of stroke than individuals with normal BMI. Furthermore, changes in the obesity phenotype, whether in BMI or metabolic status, seem to increase the risk of CVD and MI compared with the other two types of obesity. As a result, people with obesity or metabolic disorders have a higher risk of CVD (25%) and MI (18%) than people with normal weight and a healthy metabolic state (Appendix 2).

This study has some limitations. Vitamin D levels and dietary factors appear to be important contributors to CVD [34], but these variables were not assessed. Furthermore, there is a possibility of misclassification as participants may have experienced alterations in their BMI and adjustments to other CVD risk factors over the course of the follow-up period. We used self-reported data for medical history and smoking status of the participants which is prone to bias. In addition, because CVD mortality in women was rare, we were unable to apply the logistic mixed effects model to that outcome in females.

## Conclusions

It seems that modifications to metabolic disorders may have an impact on the incidence of CVD. Based on this, males with obesity and any type of metabolic disorder had a higher risk of CVD, CVD mortality, and stroke (excluding MI) compared to those with a normal BMI and no metabolic disorders. Similarly, females with obesity and any type of metabolic disorder were at higher risk of CVD, MI and stroke compared to those with a normal BMI and no metabolic disorders. This suggests that obesity and metabolic disorders are related due to their synergistic effect on high blood pressure and metabolic disorders which cause a rise in the risk of CVD. These findings confirm the importance of the prevention and early detection and treatment of obesity in children by parents, schools, and physicians. It is suggested that more studies should be conducted with larger sample sizes and more variables, especially dietary factors, to investigate the association between obesity phenotypes and CVD.

### Electronic supplementary material

Below is the link to the electronic supplementary material.

Supplementary Material 1

Supplementary Material 2

## Data Availability

The datasets generated and/or analyzed during the current study are not publicly available due to decision of the research team but are available from the corresponding author upon reasonable requests.
