# Supplementary material for: The effect of obesity phenotype changes on cardiovascular outcomes in adults older than 40 years in the prospective cohort of the Tehran lipids and glucose study (TLGS): joint model of longitudinal and time-to-event data
Source: BMC Public Health. 2024 Apr 23;24:1126. doi: 10.1186/s12889-024-18577-9 (PMC11040833; doi:10.1186/s12889-024-18577-9)
Supplement: Supplementary file 1 — Supplementary Material 1 [file 12889_2024_18577_MOESM1_ESM.docx]

**Appendix 1. Methods**

**Compilation of results**

**Data cleaning**

In the first step, data were cleaned with variables related to outcomes (CVDs, stroke, MI, and CVDs mortality). All follow-up times were checked to be recorded correctly and those times which were negative were removed. In the second step, multiple imputation was applied to participants with missing, unrecorded, or incomplete information at each phase (1). The missing values for obesity phenotypes and other variables were checked which were all under 40%. The MICE package in R was applied for imputation. Imputation has been applied five times for the purpose of detecting a more consistent dataset for non-attributed data. Sensitivity analysis was done based on hazard ratio (HR) and p-value. In the third step, the univariate analysis was performed to determine the association of each covariate (demographic variables, physical activity, smoking, and medical history of participants and their families) with the interest outcomes. Regarding that, datasets with high concurrence owing to non-imputed data were selected as underlying data.

Data collection

Subjects wearing minimal clothing and without shoes underwent weight measurement using a calibrated platform balance scale, and the results were rounded to the nearest 100 grams. Height was assessed with subjects standing, barefoot, and in a normal resting state with shoulders against a wall at three points on their bodies (buttocks, heel, and head). The height was measured and recorded in millimeters. Waist circumference (mm) was determined using a measuring tape at the midpoint between the costal margin and iliac crest along the mid-axillary line while the subjects were standing. Blood pressure was measured using an automated oscillometric sphygmomanometer. A nurse measured the blood pressure (mmHg) of participants twice, employing a properly sized blood pressure cuff with the participant's arm at heart level, after a minimum of 5 minutes of rest in a seated position. The average of the two blood pressure readings was utilized for participant categorization [12].

CVDs were broadly defined to cover any coronary heart disease events, stroke (characterized by a new neurological deficit lasting more than 24 hours), or CVD-related deaths. CHD included instances of definite MI, determined through diagnostic electrocardiogram (ECG) and biomarker results. It also included probable MI cases, which involved positive ECG findings accompanied by cardiac symptoms or signs, along with either missing biomarkers or equivocal biomarker results. Additionally, angiographically confirmed CHD and instances of CHD-related death were considered. Smoking status encompassed both past and present daily and occasional smokers. Diabetes was identified as having a FBS ≥126 mg/dl or being on antidiabetic medications. Hypertension was defined as having either a SBP ≥140 mmHg or DBP ≥90 mmHg. The body mass index (BMI) was computed by dividing the weight in kilograms by the square of the height in square meters.

**Study variables**

The primary study variables to describe participants' characteristics in the baseline included both quantitative measures including age, systolic blood pressure (SBP), diastolic blood pressure (DBP), FBS, TG, LDL, HDL, cholesterol (CHE), BMI, time to occurrence of CVDs, MI, stroke and CVDs mortality and qualitative measures including sex, level of education, physical activity, obesity phenotypes, smoking status, occurring CVDs, MI, stroke and mortality status in each phase, history of diabetes and family history of CVDs.

**Statistical analysis**

Longitudinal binary measurements, obesity phenotypes which measured in six phases of TLGS, survival times, times until occurrence of CVDs, MI, stroke and CVDs mortality, are collected for the same individuals over time. A general definition of a joint model for binary longitudinal and survival outcomes is:

P(yi (t)| bi)=1/1 + exp (XTi (t) β + ZTi (t) bi)

P(obesity phenotype)=1/1 + exp [ β_0_ +(b1* age) + (b2 * sex) + (b3*education) + (b4 * family history)]

mi (t) = E(yi (t)| bi) = g-1 (0) { XTi (t) β + ZTi (t) bi}, bi ~N (0,D)

mi (t) = E(obesity phenotype) = logit [ β_0_ +(b1* age) + (b2 * sex) + (b3*education) + (b4 * family history)]

hi (t) = h0 (t) exp [γT wi1 + ami (t)]

hi _(t)_ (CVD) = h0 (t) exp [+(b1* age) + (b2 * sex) + (b3*education) + (b4 * family history)+ (b5 * obesity phenotype) + ami (t)]

Where, y_i_ (t) is obesity phenotype at time t for subject i, i=1,2,…,n, and t =1,2,…,6, b_i_ is a random effects vector assumed to be drawn from N(0, D), X^T^_i_ (t), Z^T^_i_ (t) are the design matrix for fixed (β) and random (b) effects regression coefficients, respectively, g^-1^ (0) is the logit link function, h_0_(t) is a baseline hazard function, γ the effect of covariates on the risk of interest event, α measures the strength of the association between the risk for an event at time t and the expected value of the obesity phenotype at the same time point.

A logistic mixed effects model by including random intercepts (b_0i_) and slopes (b_1i_) for each individual allows for individual trajectories of obesity changes. The random intercept captures the baseline level of obesity for each individual, while the random slope represents how the obesity phenotype changes over time for each individual. If the random intercepts and slopes are positively correlated, it suggests that individuals who initially have a higher level of obesity tend to experience a faster rate of increase over time.

It is worth noting that the joint model was applied only for outcomes where the α parameter indicated that the association between longitudinal and time to the event model was statistically significant. If α parameter was not statistically significant, a Bayesian Cox model and a logistic mixed model were applied separately.

**References**

1. Madley-Dowd P, Hughes R, Tilling K, Heron J. The proportion of missing data should not be used to guide decisions on multiple imputation. J Clin Epidemiol. 2019 Jun;110:63-73. doi: 10.1016/j.jclinepi.2019.02.016. Epub 2019 Mar 13. PMID: 30878639; PMCID: PMC6547017.
