# Supplementary material for: The effect of obesity phenotype changes on cardiovascular outcomes in adults older than 40 years in the prospective cohort of the Tehran lipids and glucose study (TLGS): joint model of longitudinal and time-to-event data
Source: BMC Public Health. 2024 Apr 23;24:1126. doi: 10.1186/s12889-024-18577-9 (PMC11040833; doi:10.1186/s12889-024-18577-9)
Supplement: Supplementary file 2 — Supplementary Material 2 [file 12889_2024_18577_MOESM2_ESM.docx]

**Table S1.** Incidence of myocardial infarction (MI), cardiovascular diseases (CVDs), stroke, and CVD mortality in each phases among adults older than 40 years old.

|  | **CVD** | **MI** | **Stroke** | **CVD mortality** |
| --- | --- | --- | --- | --- |
| **Phases** | **N (%)** | **N (%)** | **N (%)** | **N (%)** |
| Phase 1 | 75 (1.18) | 23 (0.36) | 12 (0.19) | 15 (0.24) |
| Phase 2 | 434 (6.85) | 136 (2.15) | 56 (0.88) | 98 (1.55) |
| Phase 3 | 288 (4.62) | 78 (1.25) | 63 (1.01) | 69 (1.11) |
| Phase 4 | 293 (4.75) | 94 (1.52) | 67 (1.09) | 69(1.12) |
| Phase 5 | 269 (4.41) | 71 (1.16) | 84 (1.38) | 55 (0.90) |
| Phase 6 | 138 (2.28) | 31 (0.51) | 38 (0.63) | 29 (0.48) |

The study was conducted in six phases: phase one from 1999 to 2000, phase two from 2001 to 2004, phase three from 2005 to 2007, phase four from 2008 to 2010, phase five from 2011 to 2014, and phase six from 2015 to 2018.

| **Table S2**. Effects of time varying obesity phenotypes* and baseline covariates on incidence of MI using the joint modeling approach. | | | | | | | |
| --- | --- | --- | --- | --- | --- | --- | --- |
|  | | **MI from Bayesian Cox model** | | | **Obesity varying from Bayesian logit mixed effects model** | | |
| Variables | | Mean (SD) | HR (95% CI) | P-value | Mean (SD) | OR (95% CI) | P-value |
| Sex (Reference: Male) | Female | -1.30 (0.12) | 0.27 (0.21 to 0.35) | <0.001 | 0.49 (0.07) | 1.63 (1.40 to 1.92) | <0.001 |
| Education (Reference: illiterate) | Literate | -0.71 (0.12) | 0.49 (0.39 to 0.63) | <0.001 | - | - | - |
| Smoking status (Reference: Never smoker) | Past smoker | -0.15 (0.16) | 0.86 (0.63 to 1.15) | 0.33 | -0.03 (0.13) | 0.97 (0.75 to 1.25) | 0.82 |
|  | Current smoker | 0.14 (0.14) | 1.15 (0.84 to 1.54) | 0.31 | -0.46 (0.11) | 0.63 (0.50 to 0.79) | <0.001 |
| Physical activity (Reference: Highest) | Lowest | 0.06 (0.09) | 1.06 (0.89 to 1.27) | 0.51 | 0.22 (0.07) | 1.25 (1.08 to 1.43) | <0.001 |
| Family history of diabetes (Reference: No) | Yes | -0.02 (0.11) | 0.98 (0.79 to 1.21) | 0.82 | 0.74 (0.07) | 2.10(1.79 to 2.46) | <0.001 |
| Family history of CVD (Reference: No) | Yes | -0.01 (0.13) | 0.99 (0.76 to 1.27) | 0.93 | 0.26 (0.09) | 1.30 (1.07 to 1.58) | 0.006 |
| Obesity phenotypes (Reference: Healthy) | Unhealthy | 0.12 (0.21) | 1.13 (0.75 to 1.75) | 0.56 | - | - | - |
| Age (year) | | 0.01 (0.29) | 1.01 (0.52 to 1.49) | 0.69 | 0.02 (0.003) | 1.02 (1.02 to 1.03) | <0.001 |
| α: association between survival model and longitudinal model, p-value=0<0.001 | | | | | | | |
| Abbreviations: CVD: Cardiovascular disease; MI: Myocardial infarction SD: standard deviation; CI: credible interval; HR: hazard ratio; OR: odds ratio. | | | | | | | |
| *Metabolically unhealthy individuals, whether obese or not compared to those with metabolically healthy individuals irrespective of obesity | | | | | | | |

| **Table S3**. Effects of time varying obesity phenotypes* and baseline covariates on incidence of **stroke** using the joint modeling approach. | | | | | | | |
| --- | --- | --- | --- | --- | --- | --- | --- |
|  | | **Stroke from Bayesian Cox model** | | | **Obesity varying from Bayesian logit mixed effects model** | | |
| Variables | | Mean (SD) | HR (95% CI) | P-value | Mean (SD) | OR (95% CI) | P-value |
| Sex (Reference: Male) | Female | -0.85 (0.13) | 0.43 (0.33 to 0.56) | <0.001 | 0.49 (0.07) | 1.63 (1.39 to 1.90) | <0.001 |
| Education (Reference: illiterate) | Literate | -1.15 (0.12) | 0.32 (0.25 to 2.46) | <0.001 | - | - | - |
| Smoking status (Reference: Never smoker) | Past smoker | 0.29 (0.16) | 1.34 (0.96 to 1.82) | 0.08 | -0.04 (0.13) | 0.96 (0.74 to 1.23) | 0.74 |
|  | Current smoker | -0.20 (0.21) | 0.82 (0.53 to 1.21) | 0.33 | -0.45 (0.11) | 0.64 (0.50 to 0.79) | <0.001 |
| Physical activity (Reference: Highest) | Lowest | 0.01 (0.11) | 1.01 (0.82 to 1.25) | 0.89 | 0.23 (0.07) | 1.26 (1.08 to 1.45) | <0.001 |
| Family history of diabetes (Reference: No) | Yes | 0.21 (0.12) | 1.23 (0.97 to 1.57) | 0.08 | 0.74 (0.07) | 2.10 (1.79 to 2.44) | <0.001 |
| Family history of CVD (Reference: No) | Yes | 0.12 (0.14) | 1.13 (0.85 to 1.49) | 0.38 | 0.27 (0.09) | 1.31 (1.08 to 1.58) | 0.007 |
| Obesity phenotypes (Reference: Healthy) | Unhealthy | 0.17 (0.23) | 1.19 (0.77 to 1.86) | 0.47 | - | - | - |
| Age (year) | | 0.08 (0.17) | 1.08 (0.79 to 1.55) | 0.57 | 0.03 (0.002) | 1.03 (1.02 to 1.03) | <0.001 |
| α: association between survival model and longitudinal model, p-value=0.003 | | | | | | | |
| Abbreviations: CVD: Cardiovascular disease; SD: standard deviation; CI: credible interval ; HR: hazard ratio; OR: odds ratio. | | | | | | | |
| *Metabolically unhealthy individuals, whether obese or not compared to those with metabolically healthy individuals irrespective of obesity | | | | | | | |

| **Table S4**. Effects of time varying obesity phenotypes* and baseline covariates on incidence of **CVD** using the joint modeling approach. | | | | | | | |
| --- | --- | --- | --- | --- | --- | --- | --- |
|  | | **CVD from Bayesian Cox model** | | | **Obesity varying from Bayesian logit mixed effects model** | | |
| Variables | | Mean (SD) | HR (95% CI) | P-value | Mean (SD) | OR (95% CI) | P-value |
| Sex (Reference: Male) | Female | -0.89 (0.70) | 0.41 (0.35 to 2.12) | <0.001 | 0.48 (0.08) | 1.62 (1.36 to 1.92) | <0.001 |
| Education (Reference: illiterate) | Literate | -0.62 (0.06) | 0.54 (0.47 to 0.62) | <0.001 | - | - | - |
| Smoking status (Reference: Never smoker) | Past smoker | 0.34 (0.08) | 1.40 (1.19 to 1.67) | <0.001 | -0.05 (0.14) | 0.95 (0.37 to 1.25) | 0.69 |
|  | Current smoker | 0.10 (0.09) | 1.11 (0.92 to 1.32) | 0.27 | -0.50 (0.12) | 0.61 (0.47 to 0.78) | <0.001 |
| Physical activity (Reference: Highest) | Lowest | -0.01 (0.05) | 0.99 (0.88 to 1.08) | 0.73 | 0.24 (0.07) | 1.27 (1.49 to 1.09) | 0.001 |
| Family history of diabetes (Reference: No) | Yes | -0.02 (0.07) | 0.98 (0.86 to 1.11) | 0.73 | 0.85 (0.08) | 2.34 (1.99 to 2.75) | <0.001 |
| Family history of CVD (Reference: No) | Yes | 0.25 (0.06) | 1.28 (1.12 to 1.48) | <0.001 | - | - | - |
| Obesity phenotypes (Reference: Healthy) | Unhealthy | -0.10 (0.11) | 0.90 (0.72 to 1.14) | 0.38 | - | - | - |
| Age (year) | | -002 (0.29) | 0.98 (0.61 to 1.43) | 0.98 | 0.02 (0.004) | 1.02 (1.01 to 1.07) | <0.001 |
| α: association between survival model and longitudinal model, p-value<0.001 | | | | | | | |
| Abbreviations: CVD: Cardiovascular disease; SD: standard deviation; CI: credible interval ; HR: hazard ratio; OR: odds ratio. | | | | | | | |
| *Metabolically unhealthy individuals, whether obese or not compared to those with metabolically healthy individuals irrespective of obesity | | | | | | | |

| **Table S5.** The associations of obesity phenotype* and other study variables with incidence of **CVD mortality** using survival and longitudinal models. | | | | | |
| --- | --- | --- | --- | --- | --- |
|  | | **CVD mortality from survival model** | | **Obesity varying from logit mixed effects model** | |
| Variables | | HR (95% CI) | P-value | OR (95% CI) | P-value |
| Sex (Reference: Male) | Female | 0.58 (0.45 to 0.76) | <0.001 | 2.71 (1.84 to 3.92) | <0.001 |
| Education (Reference: illiterate) | Literate | 0.85 (0.65 to 1.11) | 0.24 | - | - |
| Family history of diabetes (Reference: No) | Yes | 1.18 (0.93 to 1.50) | 0.14 | 2.36 (1.63 to 3.42) | <0.001 |
| Family history of CVD (Reference: No) | Yes | 1.05 (0.78 to 1.40) | 0.73 | 1.18 (0.76 to 1.82) | 0.44 |
| Smoking status (Reference: Never smoker) | Past smoker | 0.57 (0.40 to 0.82) | 0.002 | 1.05 (0.65 to 1.70) | 0.83 |
|  | Current smoker | 1.35 (0.95 to 1.92) | 0.08 | 0.63 (0.37 to 1.07) | 0.09 |
| Physical activity (Reference: Highest) | Lowest | 1.05 (0.84 to 1.31) | 0.63 | 1.17 (0.84 to 1.63) | 0.33 |
| Obesity phenotypes (Reference: Healthy) | Unhealthy | 1.17 (0.88 to 1.56) | - | - | - |
| Age (year) | | 1.08 (1.07 to 1.10) | <0.001 | 1.002 (0.98 to 1.01) | <0.001 |
| Abbreviations: CVD: Cardiovascular disease; CI: credible interval; HR: hazard ratio; OR: odds ratio. | | | | | |
| *Metabolically unhealthy individuals, whether obese or not compared to those with metabolically healthy individuals irrespective of obesity | | | | | |

| **Table S6.** The associations of obesity phenotype* and other study variables with incidence of **MI** using survival and longitudinal models. | | | | | |
| --- | --- | --- | --- | --- | --- |
|  | | **MI from survival model** | | **Obesity varying from logit mixed effects model** | |
| Variables | | HR (95% CI) | P-value | OR (95% CI) | P-value |
| Sex (Reference: Male) | Female | 0.40 (0.32 to 0.50) | <0.001 | 15.12 (0.85 to 21.06) | <0.001 |
| Education (Reference: illiterate) | Literate | 0.89 (0.69 to 1.14) | 0.37 | - | - |
| Family history of diabetes (Reference: No) | Yes | 1.28 (1.04 to 1.58) | 0.01 | 2.08 (1.49 to 2.91) | <0.001 |
| Family history of CVD (Reference: No) | Yes | 1.09 (0.84 to 1.42) | 0.50 | 1.63 (1.07 to 2.48) | 0.02 |
| Smoking status (Reference: Never smoker) | Past smoker | 0.87 (0.64 to 1.19) | 0.41 | 0.63 (0.39 to 1.04) | 0.07 |
|  | Current smoker | 1.44 (0.07 to 1.92) | 0.01 | 0.27 (0.17 to 0.43) | <0.001 |
| Physical activity (Reference: Highest) | Lowest | 1.15 (0.95 to 1.39) | 0.14 | 1.65 (1.22 to 2.23) | <0.001 |
| Obesity phenotypes (Reference: Normal) | Abnormal | 1.16 (0.94 to 1.44) | 0.15 | - | - |
| Age (year) | | 1.06 (1.05 to 1.07) | <0.001 | 107 (1.05 to 1.08) | <0.001 |
| Abbreviations: CVD: Cardiovascular disease; MI: myocardial infarction: CI: credible interval; HR: hazard ratio; OR: odds ratio. | | | | | |
| * Obese BMI, regardless of healthy or unhealthy metabolic status, compared to normal BMI, regardless of healthy or unhealthy metabolic status | | | | | |

| **Table S7**. Effects of time varying obesity phenotypes* and baseline covariates on incidence of **stroke** using the joint modeling approach. | | | | | | | |
| --- | --- | --- | --- | --- | --- | --- | --- |
|  | | **Stroke from Bayesian Cox model** | | | **Obesity varying from Bayesian logit mixed effects model** | | |
| Variables | | Mean (SD) | HR (95% CI) | P-value | Mean (SD) | OR (95% CI) | P-value |
| Sex (Reference: Male) | Female | -0.30 (0.32) | 0.74 (0.41 to 1.42) | 0.36 | 2.73 (0.17) | 15.33 (10.80 to 21.54) | <0.001 |
| Education (Reference: illiterate) | Literate | -1.27 (0.29) | 0.28 (0.12 to 0.39) | <0.001 | - | - | - |
| Smoking status (Reference: Never smoker) | Past smoker | 0.28 (0.22) | 1.32 (0.9 to 2.23) | 0.15 | -0.41 (0.25) | 0.66 (0.41 to 1.08) | 0.09 |
|  | Current smoker | -0.51 (0.30) | 0.60 (0.29 to 1.01) | 0.04 | -1.24 (0.22) | 0.29 (0.19 to 0.45) | <0.001 |
| Physical activity (Reference: Highest) | Lowest | 0.17 (0.16) | 1.19 (0.97 to 1.77) | 0.24 | 0.49 (0.14) | 1.63 (1.23 to 2.18) | <0.001 |
| Family history of diabetes (Reference: No) | Yes | 0.52 (0.21) | 1.68 (1.22 to 2.94) | <0.001 | 071 (0.15) | 2.03 (1.49 to 2.72) | <0.001 |
| Family history of CVD (Reference: No) | Yes | 0.30 (0.21) | 1.35 (0.95 to 2.25) | 0.1 | 0.50 (0.19) | 1.65 (1.11 to 2.41) | 0.01 |
| Obesity phenotypes (Reference: Normal) | Abnormal | 1.40 (1.23) | 4.06 (1.15 to 9.92) | 0.01 | - | - | - |
| Age (year) | | 0.13 (0.34) | 1.14 (0.65 to 1.99) | 0.87 | 0.06 (0.005) | 1.06 (1.05 to 1.07) | <0.001 |
| α: association between survival model and longitudinal model, p-value=0.07 | | | | | | | |
| Abbreviations: CVD: Cardiovascular disease; SD: standard deviation; CI: credible interval; HR: hazard ratio; OR: odds ratio. | | | | | | | |
| * Obese BMI, regardless of healthy or unhealthy metabolic status, compared to normal BMI, regardless of healthy or unhealthy metabolic status | | | | | | | |

| **Table S8**. Effects of time varying obesity phenotypes* and baseline covariates on incidence of **CVD** using the joint modeling approach. | | | | | | | |
| --- | --- | --- | --- | --- | --- | --- | --- |
|  | | **CVD from Bayesian Cox model** | | | **Obesity varying from Bayesian logit mixed effects model** | | |
| Variables | | Mean (SD) | HR (95% CI) | P-value | Mean (SD) | OR (95% CI) | P-value |
| Sex (Reference: Male) | Female | -0.89 (0.07) | 0.41 (0.35 to 0.47) | <0.001 | 0.46 (0.08) | 1.58 (1.35 to 1.88) | <0.001 |
| Education (Reference: illiterate) | Literate | -0.62 (0.06) | 0.54 (0.47 to 0.62) | <0.001 | - | - | - |
| -Smoking status (Reference: Never smoker) | Past smoker | 0.33 (0.08) | 1.39 (1.17 to 1.65) | <0.001 | -0.04 (0.14) | 0.96 (0.73 to 1.26) | 0.73 |
|  | Current smoker | 0.11 (0.09) | 1.12 (0.93 to 1.34) | 0.23 | -0.50 (0.12) | 0.61 (0.74 to 0.78) | <0.001 |
| Physical activity (Reference: Highest) | Lowest | -002 (0.05) | 1.02 (0.88 to 1.08) | 0.71 | 0.23 (0.07) | 1.26 (1.08 to 1.46) | 0.002 |
| Family history of diabetes (Reference: No) | Yes | -0.03 (0.06) | 0.97 (0.86 to 1.09) | 0.64 | 0.82 (0.08) | 2.27 (1.93 to 2.66) | <0.001 |
| Family history of CVD (Reference: No) | Yes | 0.9 (0.07) | 1.12 (1.05 to 1.39) | 0.006 | 0.27 (0.10) | 1.31 (1.06 to 1.62) | 0.007 |
| Obesity phenotypes (Reference: Normal) | Abnormal | -0.09 (0.11) | 0.91 (0.73 to 1.14) | 0.42 | - | - | - |
| Age (year) | | 0.08 (0.21) | 1.08 (0.72 to 1.40) | 0.67 | 0.03 (0.002) | 1.02 (1.02 to 1.03) | <0.001 |
| α: association between survival model and longitudinal model, p-value<0.001 | | | | | | | |
| Abbreviations: CVD: Cardiovascular disease; SD: standard deviation; CI: credible interval; HR: hazard ratio; OR: odds ratio. | | | | | | | |
| * Obese BMI, regardless of healthy or unhealthy metabolic status, compared to normal BMI, regardless of healthy or unhealthy metabolic status | | | | | | | |

| **Table S9.** The associations of obesity phenotype* and other study variables with incidence of **CVD mortality** using survival and longitudinal models. | | | | | |
| --- | --- | --- | --- | --- | --- |
|  | | **CVD mortality from survival model** | | **Obesity varying from logit mixed effects model** | |
| Variables | | HR (95% CI) | P-value | OR (95% CI) | P-value |
| Sex (Reference: Male) | Female | 0.65 (0.50 to 0.83) | <0.001 | 18.06 (9.86 to 33.08) | <0.001 |
| Education (Reference: illiterate) | Literate | - | - | - | - |
| Family history of diabetes (Reference: No) | Yes | 1.19 (0.94 to 1.51) | 0.13 | 1.96 (1.08 to 3.54) | 0.02 |
| Family history of CVD (Reference: No) | Yes | 1.05 (0.79 to 1.41) | 0.69 | 1.26 (0.62 to 2.55) | 0.50 |
| Smoking status (Reference: Never smoker) | Past smoker | 0.56 (0.39 to 0.81) | 0.002 | 1.003 (0.43 to 2.31) | 0.99 |
|  | Current smoker | 1.33 (0.93 to 1.89) | 0.10 | 0.38 (0.15 to 0.96) | 0.04 |
| Physical activity (Reference: Highest) | Lowest | 1.05 (0.84 to 1.31) | 0.62 | 1.08 (0.62 to 1.88) | 0.76 |
| Obesity phenotypes (Reference: Normal) | Abnormal | 0.86 (0.67 to 1.10) | 0.24 | - | - |
| Age (year) | | 1.09 (1.07 to1.10) | <0.001 | 0.97 (0.94 to 0.99) | 0.02 |
| Abbreviations: CVD: Cardiovascular disease; CI: credible interval; HR: hazard ratio; OR: odds ratio. | | | | | |
| * Obese BMI, regardless of healthy or unhealthy metabolic status, compared to normal BMI, regardless of healthy or unhealthy metabolic status | | | | | |

| **Table S10.** Effects of time varying obesity phenotypes* and baseline covariates on incidence of **CVD** using the joint modeling approach. | | | | | | | |
| --- | --- | --- | --- | --- | --- | --- | --- |
|  | | **CVD from Bayesian Cox model** | | | **Obesity varying from Bayesian logit mixed effects model** | | |
| Variables | | Mean (SD) | HR (95% CI) | P-value | Mean (SD) | OR (95% CI) | P-value |
| Sex (Reference: Male) | Female | -1.24 (0.15) | 0.28 (0.21 to 0.39) | <0.001 | 2.19 (0.06) | 8.93 (8.00 to 10.07) | <0.001 |
| Education (Reference: illiterate) | Literate | -0.65 (0.06) | 0.52 (0.45 to 0.59) | <0.001 | - | - | - |
| Smoking status (Reference: Never smoker) | Past smoker | 0.42 (0.08) | 1.52 (1.30 to 1.82) | <0.001 | -0.51 (0.12) | 0.60 (0.47 to 0.78) | <0.001 |
|  | Current smoker | 0.36 (0.12) | 1.43 (1.13 to 1.82) | 0.003 | -1.70 (0.12) | 0.18 (0.15 to 0.25) | <0.001 |
| Physical activity (Reference: Highest) | Lowest | -0.03 (0.05) | 0.97 (0.86 to 1.08) | 0.56 | 0.35 (0.07) | 1.41 (1.25 to 1.63) | <0.001 |
| Family history of diabetes (Reference: No) | Yes | 0.20 (0.05) | 1.22 (1.09 to 1.35) | <0.001 | - | - | - |
| Family history of CVD (Reference: No) | Yes | 0.12 (0.07) | 1.12 (0.09 to 1.30) | 0.10 | 0.60 (0.08) | 1.82 (1.58 to 2.08) | <0.001 |
| Obesity phenotypes (Reference: Normal) | Abnormal | 0.23 (0.15) | 1.25 (0.93 to 1.72) | 0.13 | - | - | - |
| Age (year) | | 0.08 (0.12) | 1.00 (0.85 to 1.35) | 0.56 | 0.10 (0.003) | 1.10 (1.09 to 1.11) | <0.001 |
| α: association between survival model and longitudinal model, p-value<0.001 | | | | | | | |
| Abbreviations: CVD: Cardiovascular disease; SD: standard deviation; CI: credible interval; HR: hazard ratio; OR: odds ratio. | | | | | | | |
| * Obese BMI or any type of unhealthy metabolic status , compared to normal BMI and healthy metabolic status | | | | | | | |

| **Table S11.** Effects of time varying obesity phenotypes* and baseline covariates on incidence of **MI** using the joint modeling approach. | | | | | | | |
| --- | --- | --- | --- | --- | --- | --- | --- |
|  | | **MI from survival model** | | | **Obesity varying from logit mixed effects model** | | |
| Variables | | Mean (SD) | HR (95% CI) | P-value | Mean (SD) | OR (95% CI) | P-value |
| Sex (Reference: Male) | Female | -1.37 (0.14) | 0.25 (0.21 to 0.35) | <0.001 | 2.60 (0.23) | 13.46 (9.12 to 21.76) | <0.001 |
| Education (Reference: illiterate) | Literate | -0.76 (0.12) | 0.46 (0.39 to 0.63) | <0.001 | - | - | - |
| Smoking status (Reference: Never smoker) | Past smoker | -0.12 (0.15) | 0.88 (0.63 to 1.15) | 0.42 | -0.40 (0.22) | 0.67 (0.44 to 1.08) | 0.10 |
|  | Current smoker | 0.11 (0.15) | 1.11 (0.88 to 1.54) | 0.44 | -1.04 (0.22) | 0.35 (0.21 to 0.52) | <0.001 |
| Physical activity (Reference: Highest) | Lowest | 0.08 (0.09) | 1.08 (0.89 to 1.27) | 0.35 | 0.41 (0.12) | 1.50 (1.19 to 1.92) | <0.001 |
| Family history of diabetes (Reference: No) | Yes | 0.13 (0.10) | 1.13 (0.79 to 1.21) | 0.18 | - | - | - |
| Family history of CVD (Reference: No) | Yes | 0.03 (0.13) | 1.03 (0.76 to 1.27) | 0.74 | - | - | - |
| Obesity phenotypes (Reference: Normal) | Abnormal | 0.17 (0.30) | 1.18 (0.75 to 1.75) | 0.57 | - | - | - |
| Age (year) | | 0.09 (0.22) | 1.09 (0.52 to 1.49) | 0.58 | 0.08 (0.01) | 1.08 (1.07 to 1.12) | <0.001 |
| α: association between survival model and longitudinal model, p-value=0.01 | | | | | | | |
| Abbreviations: MI: myocardial infarction; CVD: Cardiovascular disease; SD: standard deviation; CI: credible interval; HR: hazard ratio; OR: odds ratio. | | | | | | | |
| * Obese BMI or any type of unhealthy metabolic status , compared to normal BMI and healthy metabolic status | | | | | | | |

| **Table S12.** The associations of obesity phenotype* and other study variables with incidence of **stroke** using survival and longitudinal models. | | | | | |
| --- | --- | --- | --- | --- | --- |
|  | | **Stroke from survival model** | | **Obesity varying from logit mixed effects model** | |
| Variables | | HR (95% CI) | P-value | OR (95% CI) | P-value |
| Sex (Reference: Male) | Female | 0.65 (0.50 to 0.84) | 0.001 | 6.00 (4.71 to 7.64) | <0.001 |
| Education (Reference: illiterate) | Literate | 0.73 (0.56 to 0.96) | 0.02 | - | - |
| Family history of diabetes (Reference: No) | Yes | 1.56 (1.24 to 1.98) | <0.001 | 2.28 (1.76 to 2.95) | <0.001 |
| Family history of CVD (Reference: No) | Yes | 1.28 (0.96 to 1.71) | 0.09 | 1.39 (1.01 to 1.92) | 0.04 |
| Smoking status (Reference: Never smoker) | Past smoker | 1.33 (0.96 to 1.85) | 0.08 | 0.65 (0.45 to 0.93) | 0.02 |
|  | Current smoker | 1.25 (0.82 to 1.90) | 0.28 | 0.29 (0.21 to 0.40) | <0.001 |
| Physical activity (Reference: Highest) | Lowest | 1.03 (0.82 to 1.29) | 0.76 | 1.39 (1.11 to 1.74) | 0.003 |
| Obesity phenotypes (Reference: Normal) | Abnormal | 1.70 (1.15 to 2.53) | 0.007 | - | - |
| Age (year) | | 1.09 (1.07 to 1.10) | <0.001 | 1.07 (1.06 to 1.07) | <0.001 |
| Abbreviations: CVD: Cardiovascular disease; CI: credible interval; HR: hazard ratio; OR: odds ratio. | | | | | |
| * Obese BMI or any type of unhealthy metabolic status, compared to normal BMI and healthy metabolic status | | | | | |

| **Table S13.** The associations of obesity phenotype* and other study variables with incidence of **CVD mortality** using survival and longitudinal models. | | | | | |
| --- | --- | --- | --- | --- | --- |
|  | | **CVD mortality from survival model** | | **Obesity varying from logit mixed effects model** | |
| Variables | | HR (95% CI) | P-value | OR (95% CI) | P-value |
| Sex (Reference: Male) | Female | 0.59 (0.45 to 0.77) | <0.001 | 7.69 (4.36 to 13.56) | <0.001 |
| Education (Reference: illiterate) | Literate | 0.85 (0.65 to 1.11) | 0.24 | - | ^-^ |
| Family history of diabetes (Reference: No) | Yes | 1.18 (0.93 to 1.50) | 0.15 | 2.65 (1.55 to 4.52) | <0.001 |
| Family history of CVD (Reference: No) | Yes | 1.04 (0.78 to 1.40) | 0.75 | 0.98 (0.54 to 1.80) | 0.92 |
| Smoking status (Reference: Never smoker) | Past smoker | 0.57 (0.40 to 0.83) | 0.003 | 0.68 (0.37 to 1.26) | 0.13 |
|  | Current smoker | 1.35 (0.95 to 1.92) | 0.09 | 0.47 (0.24 to 0.92) | 0.006 |
| Physical activity (Reference: Highest) | Lowest | 1.05 (0.85 to 1.31) | 0.62 | 1.01 (0.64 to 1.57) | 0.94 |
| Obesity phenotypes (Reference: Normal) | Abnormal | 1.17 (0.80 to 1.70) | 0.40 | - | - |
| Age (year) | | 1.08 (1.07 to 1.10) | <0.001 | 0.98 (0.95 to 1.01) | 0.29 |
| Abbreviations: CVD: Cardiovascular disease; CI: credible interval; HR: hazard ratio; OR: odds ratio. | | | |  |  |
| * Obese BMI or any type of unhealthy metabolic status, compared to normal BMI and healthy metabolic status | | | | | |
